# Supplementary material for: Sensitivity and specificity of tuberculosis screening tools in people with diabetes
Source: PLOS Glob Public Health. 2026 Sep 30;6(9):e0006479. doi: 10.1371/journal.pgph.0006479 (PMC13626337; doi:10.1371/journal.pgph.0006479)
Supplement: Supplementary file 1 [file pgph.0006479.s001.docx]

**S1 Table. Accuracy of symptom and chest X-ray screening-sensitivity analysis where trace results were considered negative**

| Screening tool | TP | FN | FP | TN | Sensitivity, % (95% CI) | Specificity, % (95% CI) | PPV, % (95% CI) | NPV, % (95% CI) |
| --- | --- | --- | --- | --- | --- | --- | --- | --- |
| Any cough | 2 | 5 | 16 | 637 | 28.6 (3.7-71.0) | 97.5 (96.1-98.6) | 11.1 (1.4-34.7) | 99.2 (98.2-99.7) |
| Any TB symptoms | 2 | 5 | 26 | 627 | 28.6 (3.7-71.0) | 96.0 (94.2-97.4) | 7.14 (0.9-23.5) | 99.2 (98.2-99.7) |
| CXR-TB suggestive abnormalities | 5 | 2 | 26 | 544 | 71.4 (29.0-96.3) | 95.4 (93.4-97.0) | 16.1 (5.5-33.7) | 99.6 (98.7-100.0) |
| CXR-Any lung abnormalities | 5 | 2 | 37 | 533 | 71.4 (29.0-96.3) | 93.5 (91.2-95.4) | 11.9 (4.0-25.6) | 99.6 (98.7-100.0) |
| CAD4TB score ≥ 60 | 3 | 3 | 12 | 519 | 50.0 (11.8-88.2) | 97.7 (96.1-98.8) | 20.0 (4.3-48.1) | 99.4 (98.3-99.9) |
| CXR-TB suggestive abnormalities and any TB symptoms | 5 | 2 | 51 | 519 | 71.4 (29.0-96.3) | 91.1 (88.4-93.3) | 8.9 (3.0-19.6) | 99.6 (98.6-100.0) |
| CXR- any lung abnormalities and any TB symptoms | 5 | 2 | 60 | 510 | 71.4 (29.0-96.3) | 89.5 (86.7-91.9) | 7.7 (2.5-17.0) | 99.6 (98.6-100.0) |

TP: true positive; FN: false negative; FP: false positive; TN: true negative; CXR: chest X-ray; CI: confidence interval: PPV: positive predictive value; NPV: negative predictive value; TB: tuberculosis
