# Supplementary material for: Sensitivity and specificity of tuberculosis screening tools in people with diabetes
Source: PLOS Glob Public Health. 2026 Sep 30;6(9):e0006479. doi: 10.1371/journal.pgph.0006479 (PMC13626337; doi:10.1371/journal.pgph.0006479)
Supplement: Supplementary file 2 [file pgph.0006479.s002.docx]

**S2 Table. Accuracy of symptom and chest X-ray screening by HIV status**

|  | **HIV-positive** | | | | | |  | **HIV-negative** | | | | | |  | p-value* |
| --- | --- | --- | --- | --- | --- | --- | --- | --- | --- | --- | --- | --- | --- | --- | --- |
| Screening tool | TP | FN | FP | TN | Sensitivity  (%) (CI 95%) | Specificity  (%) (CI 95%) |  | TP | FN | FP | TN | Sensitivity  (%) (CI 95%) | Specificity  (%) (CI 95%) |  |  |
| Any cough | 0 | 0 | 2 | 112 | NA | 98.2 (93.8-99.8) |  | 2 | 7 | 14 | 523 | 22.2 (2.8-60.0) | 97.4 (95.7-98.6) |  | 0.60 |
| Any TB symptoms | 0 | 0 | 3 | 111 | NA | 97.4 (92.5-99.5) |  | 2 | 7 | 23 | 514 | 22.2 (2.8-60.0) | 95.6 (93.6-97.3) |  | 0.42 |
| CXR-TB suggestive abnormality | 0 | 0 | 5 | 93 | NA | 94.9 (88.5-98.3) |  | 5 | 4 | 21 | 449 | 55.6 (21.2-86.3) | 95.5 (93.3-97.2) |  | 0.27 |
| CXR-Any lung abnormality | 0 | 0 | 11 | 87 | NA | 88.8 (80.8-94.3) |  | 5 | 4 | 26 | 444 | 55.6 (21.2-86.3) | 94.5 (92.0-96.4) |  | 0.04** |
| CAD4TB score ≥ 60 | 0 | 9 | 0 | 92 | NA | 100.0 (96.1-100) |  | 3 | 5 | 10 | 425 | 37.5 (8.5-75.5) | 97.7 (95.8-98.9) |  | 0.92 |
| CXR-TB suggestive abnormality and any TB symptoms | 0 | 0 | 8 | 89 | NA | 91.8 (84.4-96.4) |  | 55 | 4 | 43 | 428 | 55.6 (21.2-86.3) | 90.9 (87.9-93.3) |  | 0.78 |
| CXR- any lung abnormality and any TB symptoms | 0 | 0 | 14 | 83 | NA | 85.6 (77.0-91.9) |  | 5 | 4 | 46 | 425 | 55.6 (21.2-86.3) | 90.2 (87.2-92.8) |  | 0.18 |

TP: true positive; FN: false negative; FP: false positive; TN: true negative; CXR: chest X-ray; CI: confidence interval: PPV: positive predictive value; NPV: negative predictive value; TB: tuberculosis; NA: not available

*p-value for a difference in specificity

** p-value adjusted for previous TB history = 0.48
