# Supplementary material for: Sensitivity and specificity of tuberculosis screening tools in people with diabetes
Source: PLOS Glob Public Health. 2026 Sep 30;6(9):e0006479. doi: 10.1371/journal.pgph.0006479 (PMC13626337; doi:10.1371/journal.pgph.0006479)
Supplement: Supplementary file 3 [file pgph.0006479.s003.docx]

**S3 Table. Accuracy of symptom and chest X-ray screening by glycaemic control**

|  | **HbA1c ≥ 7.0%** | | | | | |  | **HbA1c < 7.0%** | | | | | |  | p-value* |
| --- | --- | --- | --- | --- | --- | --- | --- | --- | --- | --- | --- | --- | --- | --- | --- |
| Screening tool | TP | FN | FP | TN | Sensitivity  (%) (95%) | Specificity  (%) (CI 95%) |  | TP | FN | FP | TN | Sensitivity  (%) (CI 95%) | Specificity  (%) (CI 95%) |  |  |
| Any cough | 2 | 4 | 11 | 543 | 33.3 (4.3-77.7) | 98.0 (96.5-99.0) |  | 0 | 3 | 5 | 92 | 0 (0-70.8) | 94.8 (88.4-98.3) |  | 0.07 |
| Any TB symptoms | 2 | 4 | 21 | 533 | 33.3 (4.3-77.7) | 96.2 (94.3-97.6) |  | 0 | 3 | 5 | 92 | 0 (0-70.8) | 94.8 (88.4-98.3) |  | 0.52 |
| CXR-TB suggestive abnormality | 4 | 2 | 24 | 458 | 66.7 (22.3-95.7) | 95.0 (92.7-96.8) |  | 1 | 2 | 2 | 84 | 33.3 (0.8—90.6) | 97.7 (91.9-99.7) |  | 0.29 |
| CXR-Any lung abnormality | 4 | 2 | 33 | 449 | 66.7 (22.3-95.7) | 88.8 (80.8-94.3) |  | 1 | 2 | 4 | 82 | 33.3 (0.8—90.6) | 95.3 (88.5-98.7) |  | 0.45 |
| CAD4TB score ≥ 60 | 0 | 1 | 3 | 76 | 0.0 (0.0-70.8) | 98.7 (93.0-100) |  | 3 | 2 | 11 | 441 | 60.0 (14.7—94.7) | 97.6 (95.7-98.8) |  | 0.54 |
| CXR-TB suggestive abnormality and any TB symptoms | 4 | 2 | 44 | 438 | 66.7 (22.3-95.7) | 90.9 (87.9-93.3) |  | 1 | 2 | 7 | 79 | 33.3 (0.8—90.6) | 91.9 (83.9-96.7) |  | 0.77 |
| CXR- any lung abnormality and any TB symptoms | 4 | 2 | 52 | 430 | 66.7 (22.3-95.7) | 89.2 (86.1-91.8) |  | 1 | 2 | 8 | 78 | 33.3 (0.8—90.6) | 90.7 (82.5-95.9) |  | 0.68 |

TP: true positive; FN: false negative; FP: false positive; TN: true negative; CXR: chest X-ray; CI: confidence interval: PPV: positive predictive value; NPV: negative predictive value; TB: tuberculosis; NA: not available

*p-value for a difference in specificity
