# Supplementary material for: Sensitivity and specificity of tuberculosis screening tools in people with diabetes
Source: PLOS Glob Public Health. 2026 Sep 30;6(9):e0006479. doi: 10.1371/journal.pgph.0006479 (PMC13626337; doi:10.1371/journal.pgph.0006479)
Supplement: Supplementary file 4 [file pgph.0006479.s004.docx]

**S4 Table. Accuracy of symptom and chest X-ray screening by sex**

|  | **Female** | | | | | |  | **Male** | | | | | |  | p-value* |
| --- | --- | --- | --- | --- | --- | --- | --- | --- | --- | --- | --- | --- | --- | --- | --- |
| Screening tool | TP | FN | FP | TN | Sensitivity  (%) (95%) | Specificity  (%) (CI 95%) |  | TP | FN | FP | TN | Sensitivity  (%) (CI 95%) | Specificity  (%) (CI 95%) |  |  |
| Any cough | 2 | 2 | 10 | 407 | 50.0 (6.8-93.2) | 97.6 (95.6-98.8) |  | 0 | 5 | 6 | 228 | 0 (0-52.2) | 97.4 (94.5-99.1) |  | 0.90 |
| Any TB symptoms | 2 | 2 | 14 | 403 | 50.0 (6.8-93.2) | 96.6 (94.4-98.2) |  | 0 | 5 | 12 | 222 | 0 (0-52.2) | 94.9 (91.2-97.3) |  | 0.27 |
| CXR-TB suggestive abnormality | 3 | 1 | 11 | 349 | 75.0 (19.4-99.4) | 96.9 (94.6-98.5) |  | 2 | 3 | 15 | 193 | 40.0 (5.3-85.3) | 92.8 (88.4-95.9) |  | 0.03** |
| CXR-Any lung abnormality | 3 | 1 | 19 | 341 | 75.0 (19.4-99.4) | 94.7 (91.9-96.8) |  | 2 | 3 | 18 | 190 | 40.0 (5.3-85.3) | 91.3 (86.7-94.8) |  | 0.012*** |
| CAD4TB score ≥ 60 | 2 | 1 | 3 | 333 | 66.7 (9.4-99.2) | 99.1 (97.4-99.8) |  | 1 | 4 | 9 | 184 | 20.0 (0.5—71.6) | 95.3 (91.3-97.8) |  | 0.012 |
| CXR-TB suggestive abnormality and any TB symptoms | 3 | 1 | 25 | 335 | 75.0 (19.4-99.4) | 93.1 (89.9-95.5) |  | 2 | 3 | 26 | 182 | 40.0 (5.3-85.3) | 87.5 (82.2-91.7) |  | 0.09 |
| CXR- any lung abnormality and any TB symptoms | 3 | 1 | 32 | 328 | 75.0 (19.4-99.4) | 91.1 (87.7-93.8) |  | 2 | 3 | 28 | 180 | 40.0 (5.3-85.3) | 86.5 (81.1-90.9) |  | 0.03 |

TP: true positive; FN: false negative; FP: false positive; TN: true negative; CXR: chest X-ray; CI: confidence interval: PPV: positive predictive value; NPV: negative predictive value; TB: tuberculosis; NA: not available

*p-value for a difference in specificity

** p-value adjusted for previous TB history = 0.06

*** p-value adjusted for previous TB history = 0.23
