# Supplementary material for: Sensitivity and specificity of tuberculosis screening tools in people with diabetes
Source: PLOS Glob Public Health. 2026 Sep 30;6(9):e0006479. doi: 10.1371/journal.pgph.0006479 (PMC13626337; doi:10.1371/journal.pgph.0006479)
Supplement: Supplementary file 5 [file pgph.0006479.s005.docx]

**S5 Table. Accuracy of symptom and chest X-ray screening by previous history of TB**

|  | **Previous history of TB** | | | | | |  | **No previous history of TB** | | | | | |  | p-value* |
| --- | --- | --- | --- | --- | --- | --- | --- | --- | --- | --- | --- | --- | --- | --- | --- |
| Screening tool | TP | FN | FP | TN | Sensitivity  (%) (95%) | Specificity  (%) (CI 95%) |  | TP | FN | FP | TN | Sensitivity  (%) (CI 95%) | Specificity  (%) (CI 95%) |  |  |
| Any cough | 1 | 2 | 4 | 106 | 33.3 (0.8-90.6) | 96.4 (91.0-99.0) |  | 1 | 5 | 12 | 529 | 16.7 (0.4-64.1) | 97.8 (96.2-98.8) |  | 0.39 |
| Any TB symptoms | 1 | 2 | 7 | 103 | 33.3 (0.8-90.6) | 93.6 (87.3-97.4) |  | 1 | 5 | 19 | 522 | 16.7 (0.4-64.1) | 96.5 (94.6-97.9) |  | 0.17 |
| CXR-TB suggestive abnormality | 1 | 2 | 13 | 92 | 33.3 (0.8-90.6) | 87.6 (79.8-90.6) |  | 4 | 2 | 13 | 450 | 66.7 (22.3-95.7) | 97.2 (95.2-98.5) |  | <0.01 |
| CXR-Any lung abnormality | 1 | 2 | 16 | 89 | 33.3 (0.8-90.6) | 84.8 (76.4-91.0) |  | 4 | 2 | 21 | 442 | 66.7 (22.3-95.7) | 95.5 (93.2-97.2) |  | <0.01 |
| CAD4TB score ≥ 60 | 1 | 2 | 11 | 85 | 33.3 (0.8-90.6) | 88.5 (80.4-94.1) |  | 2 | 3 | 1 | 432 | 40.0 (5.3-85.3) | 99.8 (98.7-100) |  | <0.01 |
| CXR-TB suggestive abnormality and any TB symptoms | 1 | 2 | 19 | 85 | 33.3 (0.8-90.6) | 81.7 (72.9-88.6) |  | 4 | 2 | 32 | 432 | 66.7 (22.3-95.7) | 93.1 (90.4-95.2) |  | <0.01 |
| CXR- any lung abnormality and any TB symptoms | 1 | 2 | 22 | 82 | 33.3 (0.8-90.6) | 78.8 (69.7-86.2) |  | 4 | 2 | 38 | 426 | 66.7 (22.3-95.7) | 91.8 (88.9-94.1) |  | <0.01 |

TP: true positive; FN: false negative; FP: false positive; TN: true negative; CXR: chest X-ray; CI: confidence interval: PPV: positive predictive value; NPV: negative predictive value; TB: tuberculosis; NA: not available

*p-value for a difference in specificity
