# Supplementary material for: Sensitivity and specificity of tuberculosis screening tools in people with diabetes
Source: PLOS Glob Public Health. 2026 Sep 30;6(9):e0006479. doi: 10.1371/journal.pgph.0006479 (PMC13626337; doi:10.1371/journal.pgph.0006479)
Supplement: Supplementary file 6 [file pgph.0006479.s006.docx]

**S6 Table. Association between age and false positive results**

| **Outcome variable** | **Odds ratio (95% CI) per-10 year increase in age** | **P-value** |
| --- | --- | --- |
| Any cough | 1.45 (0.83-2.53) | 0.195 |
| Any TB symptoms | 1.02 (0.66-1.57) | 0.923 |
| CXR-TB suggestive abnormality | 1.55 (0.98-2.45) | 0.059 |
| CXR-Any lung abnormality | 1.40 (0.95-2.06) | 0.092 |
| CAD4TB score ≥ 60 | 1.37 (0.71-2.64) | 0.352 |
| CXR-TB suggestive abnormality and any TB symptoms | 1.26 (0.91-1.75) | 0.161 |
| CXR- any lung abnormality and any TB symptoms | 1.18 (0.8- 1.59) | 0.299 |

CXR: chest X-ray; CI: confidence interval: TB: tuberculosis
