# Supplementary material for: Sensitivity and specificity of tuberculosis screening tools in people with diabetes
Source: PLOS Glob Public Health. 2026 Sep 30;6(9):e0006479. doi: 10.1371/journal.pgph.0006479 (PMC13626337; doi:10.1371/journal.pgph.0006479)
Supplement: Supplementary file 8 [file pgph.0006479.s009.docx]

**Journal Requirements:**

1. Please include a complete copy of PLOS’ questionnaire on inclusivity in global research in your revised manuscript. Our policy for research in this area aims to improve transparency in the reporting of research performed outside of researchers’ own country or community. The policy applies to researchers who have travelled to a different country to conduct research, research with Indigenous populations or their lands, and research on cultural artefacts. The questionnaire can also be requested at the journal’s discretion for any other submissions, even if these conditions are not met. Please find more information on the policy and a link to download a blank copy of the questionnaire here: [https://journals.plos.org/globalpublichealth/s/best-practices-in-research-reporting](https://eur01.safelinks.protection.outlook.com/?url=https%3A%2F%2Fjournals.plos.org%2Fglobalpublichealth%2Fs%2Fbest-practices-in-research-reporting&data=05%7C02%7Cy.hamada%40ucl.ac.uk%7C3725a626cfcd40f0466f08ded1aef2de%7C1faf88fea9984c5b93c9210a11d9a5c2%7C0%7C0%7C639178746502272620%7CUnknown%7CTWFpbGZsb3d8eyJFbXB0eU1hcGkiOnRydWUsIlYiOiIwLjAuMDAwMCIsIlAiOiJXaW4zMiIsIkFOIjoiTWFpbCIsIldUIjoyfQ%3D%3D%7C0%7C%7C%7C&sdata=edTXyvapePNvgN%2FA50LrqUJ%2BItBBxwIt%2Bc0hY5EilJ0%3D&reserved=0). Please upload a completed version of your questionnaire as Supporting Information when you resubmit your manuscript.

*Response: We have uploaded the completed form.*

2. Please amend your detailed Financial Disclosure statement. This is published with the article. It must therefore be completed in full sentences and contain the exact wording you wish to be published.

i. Please clarify all sources of financial support for your study. List the grants, grant numbers, and organizations that funded your study, including funding received from your institution. Please note that suppliers of material support, including research materials, should be recognized in the Acknowledgements section rather than in the Financial Disclosure. 
ii. State the initials, alongside each funding source, of each author to receive each grant. For example: "This work was supported by the National Institutes of Health (####### to AM; ###### to CJ) and the National Science Foundation (###### to AM)."
iii. State what role the funders took in the study. If the funders had no role in your study, please state: “The funders had no role in study design, data collection and analysis, decision to publish, or preparation of the manuscript.”
iv. If any authors received a salary from any of your funders, please state which authors and which funders.

*Response: We have amended the statement accordingly.*

3. In the online submission form, you indicated that “Requests for anonymized individual‑level data underlying this study will be reviewed by CIDRI‑Africa to determine whether the request is subject to confidentiality and data protection obligations. Data that can be shared will be made available upon reasonable request and subject to a data‑sharing agreement.”.

All PLOS journals now require all data underlying the findings described in their manuscript to be freely available to other researchers, either 
1. In a public repository, 
2. Within the manuscript itself, or 
3. Uploaded as supplementary information.

This policy applies to all data except where public deposition would breach compliance with the protocol approved by your research ethics board. If your data cannot be made publicly available for ethical or legal reasons (e.g., public availability would compromise patient privacy), please explain your reasons by return email and your exemption request will be escalated to the editor for approval. Your exemption request will be handled independently and will not hold up the peer review process, but will need to be resolved should your manuscript be accepted for publication. One of the Editorial team will then be in touch if there are any issues.

*Response: We have updated the statement as follows:*

*Anonymized data underlying the analyses reported in this article are available at: https://doi.org/10.6084/m9.figshare.33107255*

4. Please ensure that your Ethics Statement is available in its entirety at the beginning of your Methods section, under a subheading 'Ethics Statement'. It must include:

i) The name(s) of the Institutional Review Board(s) or Ethics Committee(s)

ii) The approval number(s), or a statement that approval was granted by the named board(s)

iii) (for human participants or donors) - A statement that formal consent was obtained (must state whether verbal/written) OR the reason consent was not obtained (e.g., anonymity).

vi) (for child participants) - Please update your Ethics Statement with confirmation that written informed consent was obtained from the parent/guardian of each participant under 18 years of age.

*Response: We have updated the Ethics Statement accordingly. We would also like to clarify that, although the protocol permitted enrolment of participants aged 15–17 years, no individuals under 18 years of age were screened or enrolled. Therefore, parental or guardian consent was not required. We have updated the text of the manuscript to clarify:*

*“Individuals aged 15 years and older with diabetes were eligible; however, all participants screened and enrolled were aged 18 years or older.”*

5. We have noticed that you have uploaded Supporting Information files, but you have not included a list of legends. Please add a full list of legends for your Supporting Information files after the references list.

*Response: We have updated the manuscript accordingly.*

**Reviewer #1**: Manuscript Number: PGPH-D-26-01594

1. For the study, you used a manufacturer’s cut-off for the CAD4 TB score, which might be inappropriate for your population. Using your data, are you able to compute a cut-off ideal for this local population, because AI-aided diagnosis of TB is the future of TB diagnosis in high TB burden settings?

*Response: Thank you for this comment. We agree that CAD4TB thresholds may require local calibration. However, only eight participants with TB had evaluable CAD4TB scores, so our dataset is too small to derive a reliable population-specific cut-off. We therefore retained the prespecified manufacturer-recommended threshold of 60 and clarified that larger studies are needed to identify and validate an appropriate threshold for people with diabetes in this setting.*

*“Second, our dataset was not sufficiently powered to derive or validate a population-specific threshold. Larger studies are needed to identify and validate an appropriate threshold for people with diabetes in this setting.”*

2. In the discussion section, could you include plausible theories or hypotheses to explain the differences in the sensitivity of CXR readings in people with poor vs good glycaemic control and males vs women?

*Response: Thank you for this comment. Although point estimates of chest X-ray sensitivity differed by glycaemic control and sex, these analyses were based on very small numbers of TB cases, and the confidence intervals were wide and overlapping. We therefore considered that proposing biological explanations would risk overinterpreting the findings. Accordingly, we have revised the Results to report the subgroup estimates without suggesting that differences were observed and have emphasised their imprecision.*

*“The sensitivity of chest X-ray among those with poor glycaemic control (HbA1c ≥7.0%) was 66.7% (95% CI: 22.3–95.7%) and 33.0% (95% CI: 0.8–90.6%) among those with better control (HbA1c < 7.0%. The corresponding estimates were 75.0% (95% CI: 19.4–99.4%) in females and 40.0% (95% CI: 5.3–85.3%) in males. In both comparisons, the confidence intervals were wide and overlapping.”*

3. In the discussion section, in addition to using AI-aided technology, could you propose additional strategies that can be used in clinical practice or explored in research to improve the diagnosis of TB in people living with diabetes? For example, is there a potential role of haematological parameters such as absolute WBC counts, neutrophil: lymphocyte ratio, and monocyte: lymphocyte ratio? These are simple and cheap parameters that have been shown to predict specific TB treatment outcomes.

*Response: Thank you for this suggestion. We note that C-reactive protein warrants further evaluation in this population. We have also added universal molecular testing, irrespective of symptoms or chest X-ray findings, as a potential strategy to improve TB case detection.*

*“The use of additional tools, such as C-reactive protein, may warrant exploration,[18] but data on its utility among people with diabetes are currently unavailable. Universal molecular testing, irrespective of symptoms or chest X-ray findings, may also improve TB case detection; however, its feasibility and cost-effectiveness require evaluation.”*

**Reviewer #2:** PLOS Global Public Health

Comment one: The authors could improve the description of the diabetic population phenotype in this study. How many participants had other comorbidites other than HIV? What diabetic medications were the participants on? Especially in the light that some diabetic medications can slow TB progression or precipate it? How many of the patients with HIV had a hsitory of taking TB preventive therapy? What about the socio-economic status? Levels of education? Duration of the diabetes.

*Response: We have revised Table 1 to include time since diabetes diagnosis, use of diabetes medications (metformin and insulin), and comorbidities (hypertension and chronic kidney disease). Unfortunately, other variables, including education, socioeconomic status, and history of TB preventive treatment, were not available.*

Comment two: We appreciate that all participants gave sputum for TB evaluation. How many presented with any cough? A description of the sputum collection or induction process in the methods section would be good.

*Response: Thank you for the questions.*

*We have added: “Any cough was reported by 18 participants (2.7%), and any TB symptom by 28 participants (4.2%).”*

*We also added:*

What were the different cough durations. This population often has respiratory infections, especially viral respiratory infections. The question would arise if we need to evaluate such coughs for TB and the cost implications eventually. Therefore a sub-analysis based on duration would be very insightful.

*Response: Thank you for this suggestion. Unfortunately, we did not collect data on cough duration and were therefore unable to undertake this analysis.*

Comment three: It would also be helpful to also describe the prevalence of the TB symptoms assessed. And also the proportions of the CAD4TB scores below or above 60.

*Response:We have updated the Results to report the prevalence of TB symptoms and the proportion of participants with CAD4TB scores above the threshold of 60, as follows:*

*“Any cough was reported by 18 participants (2.7%), and any TB symptom by 28 participants (4.2%). Chest X-ray results were missing for 81 participants without a TB diagnosis. Among the 580 participants with chest X-ray results, CAD4TB scores were missing for 43 participants (7.4%), including 42 without TB and one with TB. Of the 537 participants with available CAD4TB scores, 15 (2.8%) had a score ≥60.”*

In the combined classification, were all TB symptoms weighted equally?
*Response: Yes. In accordance with the WHO symptom-screening definition, the presence of any one of the four symptoms was considered a positive screen; no differential weighting was applied.*

Comment four: Where was the TB testing performed? At the peripheral Health center TB laboratory or at TB accredited BSL 3 laboratory?

*Response:*

*Thank you for this comment. All sputum samples were processed and tested under biosafety level 3 conditions at a University of Cape Town research laboratory. We have added this information to the Methods.*

However, the limitations of using a positive Xpert MTB/RIF Ultra test result as the reference standard instead of sputum culture are noted.

*Response: Thank you for acknowledging that this limitation has been addressed in the Discussion.*

Comment five: There were 17.2% participants with previous TB in the study. Why did the authors include the chest X-rays (CXRs) of these participants in the analysis? The results are not surprising and therefore another analysis excluding this population might have better appreciated results.

*Response: Thank you for this comment. We included participants with previous TB because previous TB is common in high-burden settings and these individuals form part of the population encountered in routine TB screening. Note that WHO guidance does not specify that individuals with a history of TB should be excluded from CXR-based screening. Excluding them could reduce the generalisability of our findings and overestimate the specificity of CXR-based screening. We addressed the potential effect of previous TB through a prespecified stratified analysis according to history of previous TB.*

Comment six: The study adjusted for most of the confounders but omitted occupation which in South Africa is an important risk factor. How many of these participants were miners or have risky occupations? This could affect the CXR findings.

*Response: Thank you for this comment. Occupation and occupational exposure history were not collected; therefore, we were unable to determine how many participants had worked in mining or other high-risk occupations or assess their potential influence on chest X-ray findings.*

Comment seven: How do these findings align or misalign to finding in other vulnerable populations or to the national TB screening algorithm?

*Response: Thank you for this comment. The Discussion already compares our findings with sensitivity estimates reported in the WHO systematic screening guidelines and in other populations. Although our findings are consistent with the generally poor sensitivity of symptom-based screening, the estimated sensitivity of both symptom screening and chest X-ray was lower than that reported elsewhere. However, these estimates were imprecise, with wide confidence intervals.*

Comment eight: The authors did not discuss all the results despite having many supplementary results depriving the readership of deeper explorations of the findings. And they only referenced 18 studies for such an important study.

*Response:* *Thank you for this comment. We have revised the Discussion to address the supplementary analyses more fully, including the findings according to glycaemic control, sex, age, HIV status, and previous TB. We now highlight that specificity was lower among participants with previous TB but remained consistent across the other clinical factors assessed, which is reassuring.* *We also reviewed the reference list and confirmed that the key relevant literature is cited; no additional references were considered necessary.*

*“Our study found a significantly lower specificity of chest X-ray abnormalities in individuals with a history of TB compared to those without. This is likely due to persistent chest X-ray abnormalities from previous TB, leading to false positives. On the other hand, the specificity of the screening tools remained consistent across other clinical factors, including glycaemic control, sex, age, and HIV status, which is reassuring. However, the impact of these clinical factors on sensitivity remains unclear and requires further evaluation.”*
