# Supplementary material for: Sensitivity and specificity of tuberculosis screening tools in people with diabetes
Source: PLOS Glob Public Health. 2026 Sep 30;6(9):e0006479. doi: 10.1371/journal.pgph.0006479 (PMC13626337; doi:10.1371/journal.pgph.0006479)
Supplement: Peer review history

# Peer review: Decision Letter 0

**DOI:** 10.1371/journal.pgph.0006479.r001

**Authors:** Joseph Baruch Baluku

23 Jun 2026

PGPH-D-26-01594

Sensitivity and specificity of tuberculosis screening tools in people with diabetes

PLOS Global Public Health

Dear Dr. HAMADA,

Thank you for submitting your manuscript to PLOS Global Public Health. After careful consideration, we feel that it has merit but does not fully meet PLOS Global Public Health’s publication criteria as it currently stands. Therefore, we invite you to submit a revised version of the manuscript that addresses the points raised during the review process.

Please submit your revised manuscript by Aug 22 2026 11:59PM. If you will need more time than this to complete your revisions, please reply to this message or contact the journal office at globalpubhealth@plos.org. When you're ready to submit your revision, log on to https://www.editorialmanager.com/pgph/ and select the 'Submissions Needing Revision' folder to locate your manuscript file.

Please include the following items when submitting your revised manuscript:

- A letter that responds to each point raised by the editor and reviewer(s). You should upload this letter as a separate file labeled 'Response to Reviewers'.
- A marked-up copy of your manuscript that highlights changes made to the original version. You should upload this as a separate file labeled 'Revised Manuscript with Track Changes'.
- An unmarked version of your revised paper without tracked changes. You should upload this as a separate file labeled 'Manuscript'.

Guidelines for resubmitting your figure files are available below the reviewer comments at the end of this letter.

As the corresponding author, your ORCID iD is verified in the submission system and will appear in the published article. PLOS supports the use of ORCID, and we encourage all coauthors to register for an ORCID iD and use it as well. Please encourage your coauthors to verify their ORCID iD within the submission system before final acceptance, as unverified ORCID iDs will not appear in the published article. *Only*  the individual author can complete the verification step; PLOS staff *cannot*  verify ORCID iDs on behalf of authors.

We look forward to receiving your revised manuscript.

Kind regards,

Joseph Baruch Baluku, MMed

Academic Editor

PLOS Global Public Health

**Journal Requirements:**

1. Please include a complete copy of PLOS’ questionnaire on inclusivity in global research in your revised manuscript. Our policy for research in this area aims to improve transparency in the reporting of research performed outside of researchers’ own country or community. The policy applies to researchers who have travelled to a different country to conduct research, research with Indigenous populations or their lands, and research on cultural artefacts. The questionnaire can also be requested at the journal’s discretion for any other submissions, even if these conditions are not met. Please find more information on the policy and a link to download a blank copy of the questionnaire here: https://journals.plos.org/globalpublichealth/s/best-practices-in-research-reporting. Please upload a completed version of your questionnaire as Supporting Information when you resubmit your manuscript.

2. Please amend your detailed Financial Disclosure statement. This is published with the article. It must therefore be completed in full sentences and contain the exact wording you wish to be published.

i. Please clarify all sources of financial support for your study. List the grants, grant numbers, and organizations that funded your study, including funding received from your institution. Please note that suppliers of material support, including research materials, should be recognized in the Acknowledgements section rather than in the Financial Disclosure.

ii. State the initials, alongside each funding source, of each author to receive each grant. For example: "This work was supported by the National Institutes of Health (####### to AM; ###### to CJ) and the National Science Foundation (###### to AM)."

iii. State what role the funders took in the study. If the funders had no role in your study, please state: “The funders had no role in study design, data collection and analysis, decision to publish, or preparation of the manuscript.”

iv. If any authors received a salary from any of your funders, please state which authors and which funders.

3. In the online submission form, you indicated that “Requests for anonymized individual‑level data underlying this study will be reviewed by CIDRI‑Africa to determine whether the request is subject to confidentiality and data protection obligations. Data that can be shared will be made available upon reasonable request and subject to a data‑sharing agreement.”.

All PLOS journals now require all data underlying the findings described in their manuscript to be freely available to other researchers, either

1. In a public repository,

2. Within the manuscript itself, or

3. Uploaded as supplementary information.

This policy applies to all data except where public deposition would breach compliance with the protocol approved by your research ethics board. If your data cannot be made publicly available for ethical or legal reasons (e.g., public availability would compromise patient privacy), please explain your reasons by return email and your exemption request will be escalated to the editor for approval. Your exemption request will be handled independently and will not hold up the peer review process, but will need to be resolved should your manuscript be accepted for publication. One of the Editorial team will then be in touch if there are any issues.

4. Please ensure that your Ethics Statement is available in its entirety at the beginning of your Methods section, under a subheading 'Ethics Statement'. It must include:

i) The name(s) of the Institutional Review Board(s) or Ethics Committee(s)

ii) The approval number(s), or a statement that approval was granted by the named board(s)

iii) (for human participants or donors) - A statement that formal consent was obtained (must state whether verbal/written) OR the reason consent was not obtained (e.g., anonymity).

vi) (for child participants) - Please update your Ethics Statement with confirmation that written informed consent was obtained from the parent/guardian of each participant under 18 years of age.

5. We have noticed that you have uploaded Supporting Information files, but you have not included a list of legends. Please add a full list of legends for your Supporting Information files after the references list.

If the reviewer comments include a recommendation to cite specific previously published works, please review and evaluate these publications to determine whether they are relevant and should be cited. There is no requirement to cite these works unless the editor has indicated otherwise.

**Additional Editor Comments (if provided):**

[Note: HTML markup is below. Please do not edit.]

Reviewers' comments:

Reviewer's Responses to Questions

**Comments to the Author**

1. Does this manuscript meet PLOS Global Public Health’s publication criteria?

Reviewer #1: Yes

Reviewer #2: Yes

**********

2. Has the statistical analysis been performed appropriately and rigorously?

Reviewer #1: Yes

Reviewer #2: I don't know

**********

3. Have the authors made all data underlying the findings in their manuscript fully available (please refer to the Data Availability Statement at the start of the manuscript PDF file)?

The PLOS Data policy

Reviewer #1: Yes

Reviewer #2: Yes

**********

4. Is the manuscript presented in an intelligible fashion and written in standard English?

Reviewer #1: Yes

Reviewer #2: Yes

**********

**Reviewer #1** : Manuscript Number: PGPH-D-26-01594

Title: Sensitivity and specificity of tuberculosis screening tools in people with diabetes

Dear Editor, many thanks for requesting me to review this manuscript by Janrode et al.

It is a well-written paper addressing an important public health challenge in a clinical setting with a high dual TB-diabetes burden.

Below are my minor comments.

1. For the study, you used a manufacturer’s cut-off for the CAD4 TB score, which might be inappropriate for your population. Using your data, are you able to compute a cut-off ideal for this local population, because AI-aided diagnosis of TB is the future of TB diagnosis in high TB burden settings?

2. In the discussion section, could you include plausible theories or hypotheses to explain the differences in the sensitivity of CXR readings in people with poor vs good glycaemic control and males vs women?

3. In the discussion section, in addition to using AI-aided technology, could you propose additional strategies that can be used in clinical practice or explored in research to improve the diagnosis of TB in people living with diabetes? For example, is there a potential role of haematological parameters such as absolute WBC counts, neutrophil: lymphocyte ratio, and monocyte: lymphocyte ratio? These are simple and cheap parameters that have been shown to predict specific TB treatment outcomes.

**Reviewer #2:**  PLOS Global Public Health

Sensitivity and specificity of tuberculosis screening tools in people with diabetes

Manuscript Number: PGPH-D-26-01594

Article Type: Research Article

REVIEWERS COMMENTS

This is an important area of study.

Comment one: The authors could improve the description of the diabetic population phenotype in this study. How many participants had other comorbidites other than HIV? What diabetic medications were the participants on? Especially in the light that some diabetic medications can slow TB progression or precipate it? How many of the patients with HIV had a hsitory of taking TB preventive therapy? What about the socio-economic status? Levels of education? Duration of the diabetes.

Comment two: We appreciate that all participants gave sputum for TB evaluation. How many presented with any cough? A descritpion of the sputum collection or induction process in the methods section would be good.

What were the different cough durations. This population often has respiratory infections, especially viral respiratory infections. The question would arise if we need to evaluate such coughs for TB and the cost implications eventually. Therefore a sub-analysis based on duration would be very insightful.

Comment three: It would also be helpful to also describe the prevalence of the TB symptoms assessed. And also the proportions of the CAD4TB scores below or above 60.

In the combined classification, were all TB symptoms weighted equally?

Comment four: Where was the TB testing performed? At the peripheral Health center TB laboratory or at TB accredited BSL 3 laboratory?

However, the limitations of using a positive Xpert MTB/RIF Ultra test result as the reference standard instead of sputum culture are noted.

Comment five: There were 17.2% participants with previous TB in the study. Why did the authors include the chest X-rays (CXRs) of these participants in the analysis? The results are not surprising and therefore another analysis excluding this population might have better appreciated results.

Comment six: The study adjusted for most of the confounders but omitted occupation which in South Africa is an important risk factor. How many of these participants were miners or have risky occupations? This could affect the CXR findings.

Comment seven: How do these findings align or misalign to finding in other vulnerable populations or to the national TB screening algorithm?

Comment eight: The authors did not discuss all the results despite having many supplementary results depriving the readership of deeper explorations of the findings. And they only referenced 18 studies for such an important study.

**********

what does this mean?). If published, this will include your full peer review and any attached files.

**Do you want your identity to be public for this peer review?**  If you choose “no”, your identity will remain anonymous but your review may still be made public.

For information about this choice, including consent withdrawal, please see our Privacy Policy

Reviewer #1: No

Reviewer #2: No

**********

[NOTE: If reviewer comments were submitted as an attachment file, they will be attached to this email and accessible via the submission site. Please log into your account, locate the manuscript record, and check for the action link "View Attachments". If this link does not appear, there are no attachment files.]

**Figure Resubmissions:**

# Author comment: Author response to Decision Letter 1

**DOI:** 10.1371/journal.pgph.0006479.r002

30 Jul 2026

Attachment Submitted filename: Response to reviewers.docx

# Editor report: Decision Letter 1

**DOI:** 10.1371/journal.pgph.0006479.r003

**Authors:** Joseph Baruch Baluku

4 Aug 2026

Sensitivity and specificity of tuberculosis screening tools in people with diabetes

PGPH-D-26-01594R1

Dear Dr HAMADA,

We are pleased to inform you that your manuscript 'Sensitivity and specificity of tuberculosis screening tools in people with diabetes' has been provisionally accepted for publication in PLOS Global Public Health.

Before your manuscript can be formally accepted you will need to complete some formatting changes, which you will receive in a follow up email. A member of our team will be in touch with a set of requests.

Please note that your manuscript will not be scheduled for publication until you have made the required changes, so a swift response is appreciated.

IMPORTANT: The editorial review process is now complete. PLOS will only permit corrections to spelling, formatting or significant scientific errors from this point onwards. Requests for major changes, or any which affect the scientific understanding of your work, will cause delays to the publication date of your manuscript.

If your institution or institutions have a press office, please notify them about your upcoming paper to help maximize its impact. If they'll be preparing press materials, please inform our press team as soon as possible -- no later than 48 hours after receiving the formal acceptance. Your manuscript will remain under strict press embargo until 2 pm Eastern Time on the date of publication. For more information, please contact globalpubhealth@plos.org.

Thank you again for supporting Open Access publishing; we are looking forward to publishing your work in PLOS Global Public Health.

Best regards,

Joseph Baruch Baluku, MMed

Academic Editor

PLOS Global Public Health

***********************************************************

None.

Reviewer Comments (if any, and for reference):
